# A Systematic Review of COVID-19 Vaccine-Induced Takotsubo Cardiomyopathy: A 2023 Update

**DOI:** 10.7759/cureus.50319

**Published:** 2023-12-11

**Authors:** Binayak Singh, Bai Manita, FNU Suman, Nikita Kumari, Saher T Shiza, Iqra Samreen, Siddhi Shah, Saria M Mokhtar, Utsav Patel, Joti Devi, Rezaur Rahman Reza, Khalid H Mohamed, Sarfaraz Ahmad, Hira Nasir

**Affiliations:** 1 Medicine, Mayo Hospital, Lahore, PAK; 2 Internal Medicine, Ghulam Muhammad Mahar Medical College, Sukkur, PAK; 3 Internal Medicine, NYC Health and Hospitals-Lincoln Hospital, New York, USA; 4 Medicine, Deccan College of Medical Sciences, Hyderabad, IND; 5 Medicine and Surgery, Hinduhridaysamrat Balasaheb Thackeray (HBT) Medical College and Dr. RN (Rustom Narsi) Cooper Municipal General Hospital, Mumbai, IND; 6 Research, California Institute of Behavioral Neurosciences & Psychology, Fairfield, USA; 7 Internal Medicine, Medical College Baroda and SSG (Sir Sayajirao General) Hospital, Vadodara, IND; 8 Pharmacy, Clifton Medical Services, Karachi, PAK; 9 Internal Medicine, Jalalabad Ragib Rabeya Medical College, Sylhet, BGD; 10 Neurology, Sheffield Teaching Hospitals NHS Foundation Trust, Sheffield, GBR; 11 Internal Medicine, Saint James School of Medicine, Chicago, USA; 12 Internal Medicine, Mayo Hospital, Lahore, PAK

**Keywords:** broken-heart syndrome, stress induced cardiomyopathy, takotsubo cardiomyopathy (ttc), covid-19 vaccine complication, covid-19 vaccine

## Abstract

Takotsubo cardiomyopathy (TCM) is a life-threatening transient left ventricular dysfunction triggered by either physical or emotional stressors. Concerns have been raised on reports of TCM after the coronavirus disease 2019 (COVID-19) vaccine. Our study provides comprehensive detail on COVID-19 vaccine-induced TCM.

We conducted a systemic literature search using major databases, including PubMed, EMBASE, and Google Scholar up to November 2023, to identify cases of COVID-19 vaccine-induced TCM using the MeSH terms and keywords "covid-19 vaccines" and "takotsubo cardiomyopathy".

We identified 15 case reports, including 16 patients with COVID-19 vaccine-induced TCM. The mean age was 55.81 ± 19.13 years, and 75% of the patients were female. The most common presentation was chest pain (62.5%), and the average time to first symptom onset was 3.12 ± 2.24 days. COVID-19 vaccine-induced TCM was reported in 43.75% of patients receiving the first and second dose each, and 87% of patients had messenger ribonucleic acid (mRNA) COVID-19 vaccine (Pfizer, Moderna). The elevated level of cardiac troponins was found in all the patients with a left ventricular ejection fraction (LVEF) of <50% in 15 patients, and T-wave inversion (50%) was the most common electrocardiographic finding. The mean length of the hospital stay was 7.27 ± 3.95 days, and 87% of patients were discharged.

COVID-19 vaccine-induced TCM is a rare but life-threatening complication. TCM should be included in the differential diagnosis of chest pain or dyspnea in patients recently receiving the COVID-19 vaccine.

## Introduction and background

Recently, the global response to the coronavirus disease 2019 (COVID-19) pandemic has been marked by the rapid development and deployment of various vaccines to mitigate the spread and severity of the virus [1]. While the vaccines have demonstrated remarkable efficacy in preventing severe illness and death associated with COVID-19, the scientific and medical communities continually evaluate and monitor potential adverse effects to ensure the safety of vaccination programs [2]. Vaccines developed against COVID-19 include messenger ribonucleic acid (mRNA) vaccines (Pfizer-BioNTech (Mainz, Germany) and Moderna (Massachusetts, United States)), viral vector vaccines (AstraZeneca (Cambridge, United Kingdom), Johnson & Johnson's Janssen (New Jersey, United States), and Sputnik V (The Gamaleya National Center of Epidemiology and Microbiology, Moscow, Russia)), and protein subunit vaccines (Novavax (Maryland, USA)) [3]. Although each vaccine has demonstrated its efficacy in COVID-19 prevention, no vaccine is free from any side effects or complications. Vaccine-induced side effects or complications generally depend on the type of vaccine and the dosing of the vaccine [4]. Commonly reported side effects include injection site redness, swelling, pain, fatigue, headache, nausea, myalgia, or fever (Table 1) [5].

**Table 1:** Commonly reported side effects of COVID-19 vaccine Source: [5]

Side effect	Number of patients	Percentage (%)
Injection site pain	851	78.4
Injection site swelling	177	16.3
Fever	300	27.6
Headache	359	33.1
Myalgia	408	37.6
Fatigue	563	51.8
Nausea	164	15.1

COVID-19 vaccine has also been reported to involve any organ system of the body and vaccine-induced cardiovascular, neurological, and gastrointestinal system complications have also been reported [6]. Myocarditis, pericarditis, myocardial infarction, myocardial injury, coagulopathy, heart failure, and arrhythmias are reported cardiovascular complications of COVID-19 vaccine [7]. Takotsubo cardiomyopathy (TCM) is also an uncommon, life-threatening complication of the COVID-19 vaccine, especially with mRNA COVID-19 vaccines (Pfizer-BioNTech and Moderna) [8].

TCM is a sudden, transient heart syndrome that mimics acute coronary syndrome. It is also referred to as broken heart syndrome or stress cardiomyopathy, characterized by left ventricular apical akinesia [9]. This condition typically occurs following intense emotional or physical stress such as the death of a loved one, a traumatic event, or a severe illness [9]. The association between the COVID-19 vaccine and the development of TCM has not been widely reported, and only a limited number of cases have been reported [10]. Our study aims to consolidate and analyze the evidence regarding COVID-19 vaccine-induced TCM.

## Review

Material and methods

**Figure 1:**
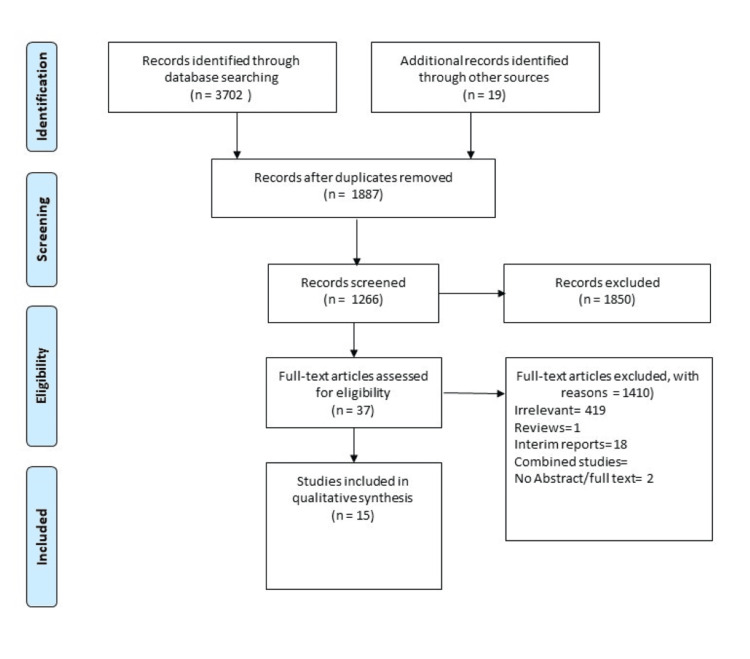
PRISMA flow diagram PRISMA: Preferred Reporting Items for Systematic Review and Meta-Analyses

A systematic and comprehensive literature search was conducted using electronic databases, including PubMed, MEDLINE, Embase, and Cochrane Library, in accordance with the Preferred Reporting Items for Systematic Review and Meta-Analyses (PRISMA) guidelines [11] (Figure 1). Our data search included the Medical Subject Headings (MeSH) terms and keywords for the COVID-19 vaccine and takotsubo cardiomyopathy from the date of inception to November 19, 2023. We did not limit our search to any language or geographical area. Our search also included the references of the relevant articles identifying all the pertinent studies, and all citations were downloaded to EndNote version 12.0 (https://endnote.com/downloads/available-updates/) for further screening and extraction of related data. We stratified only those studies reporting COVID-19 vaccine-inducing TCM regardless of the type and dosing. All those studies with a lack of COVID-19-induced TCM data were excluded.

Our initial search revealed 3721 articles; two authors screened titles and abstracts of identified articles based on inclusion and exclusion criteria. Full-text articles of potentially relevant studies were then assessed for eligibility. All included studies were evaluated independently by two authors using the critical appraisal tool of the Joanna Briggs Institute for case reports. A pre-defined Microsoft Excel data sheet (Microsoft Corporation, Redmond, WA) was used to extract data. Relevant data were extracted from eligible studies, including study design, participant demographics, type of COVID-19 vaccine administered, time to onset of takotsubo cardiomyopathy, dose of the vaccine, length of hospital stay, electrocardiographic (ECG) changes, ejection fraction (%), treatment, and clinical outcomes. We expressed the categorical data as proportions (%) and the numerical data as mean and standard deviation (SD) using the Statistical Package for the Social Sciences (SPSS) 24.0 (IBM Corp., Redmond, WA). Data extraction was performed independently by two reviewers, with discrepancies resolved through consensus. The quality of the systematic review was assessed using the A Measurement Tool to Assess Systematic Reviews 2 (AMSTAR 2) tool [12].

Results

Out of 3721 articles, 31 studies were assessed for eligibility, and 15 case reports were included in our study, reporting 16 patients developing TCM following the COVID-19 vaccine. Characteristics of each study, including demographics, type of the vaccine, dosage of the vaccine, onset of symptoms following vaccination, manifestation of TCM at presentation, electrocardiographic changes, ejection fraction, length of the hospital stay, and the management and outcome of each patient are shown in Table 2 [8,10,13-25].

**Table 2:** Characteristics of reported cases of COVID-19 vaccine-induced TCM TCM: takotsubo cardiomyopathy, LVEF: left ventricular ejection fraction, M: male, NR: not reported, F: female, ECG: electrocardiography, mRNA: messenger ribonucleic acid Source: [8,10,13-25]

Author/Year	Age (years)/Sex	Type of vaccine	Dose of vaccine	Days to symptom onset (days)	Symptoms	Troponin level	LVEF (%)	ECG	Hospital stays (day)	Management	Outcome
Reza et al., 2023 [8]	59/F	mRNA-1273	3^rd^	3	Dyspnea	Elevated	< 50	Abnormal	7	Furosemide, hydrocortisone, norepinephrine	Improved
Minicullo et al., 2023 [10]	54/F	BNT162b2	1st	1	Dyspnea, confusion	Normal	< 50	Abnormal	4	Oxygen, steroids, diuretics	Improved
Beshai et al., 2022 [13]	45/M	mRNA-1273	2^nd^	3	Chest discomfort	Elevated	< 50	Abnormal	4	NA	Improved
Chen et al., 2023 [14]	67/F	mRNA-1273	1^st^	7	Palpitation, chest discomfort	Elevated	< 50	Abnormal	7	Bisoprolol, steroids, diuretics	Improved
Yamaura et al., 2022 [15]	30/F	BNT162b2	2^nd^	2	Cold sweat, chest discomfort	Elevated	< 50	Abnormal	15	The patient was managed without medical therapies	Improved
Tedeschi et al., 2022 [16]	71/F	BNT162b2	1^st^	1	Dyspnea, chest discomfort	Elevated	< 50	Abnormal	NR	NR	Improved
Gill et al., 2022 [17]	18/M	BNT162b2	2^nd^	3	Asymptomatic	Elevated	< 50	Abnormal	3	NR	Death
Gill et al., 2022 [17]	18/M	BNT162b2	2nd	4	Asymptomatic	Elevated	< 50	Abnormal	4	NR	Death
Boscolo et al., 2021 [18]	63/F	mRNA-1273	1^st^	1	Dyspnea, fever	Elevated	< 50	Abnormal	NR	NR	Improved
Jani et al., 2021 [19]	65/F	mRNA-1273	1^st^	1	Headache, nausea, muscle ache, chest discomfort	Elevated	< 50	Abnormal	NR	Dual antiplatelet, metoprolol succinate,	Improved
Toida et al., 2021 [20]	80/F	BNT162b2	1^st^	1	Anorexia, generalized fatigue	Elevated	< 50	Abnormal	13	Intravenous fluid, oxygen therapy	Improved
Stewart et al., 2021 [21]	50/F	ChadOX1 nCOV-19	2^nd^	7	Nausea, chest discomfort	Elevated	< 50	Abnormal	5	Oxygen therapy, dual antiplatelet	Improved
Fearon et al., 2021 [22]	73/F	mRNA-1273	NR	1	Nausea, chest discomfort, dyspnea	Elevated	> 50	Abnormal	8	Diuretics, antiplatelet therapy, metoprolol	Improved
Wardhere et al., 2021 [23]	68/F	mRNA-1273	2^nd^	7	Chest discomfort	Elevated	< 50	Abnormal	NR	NR	Improved
Crane et al., 2021 [24]	72/M	ChadOX1 nCOV-19	1^st^	4	Chest pain, fatigue, myalgias, fever	Elevated	< 50	Abnormal	10	Dual antiplatelet therapy, losartan, metoprolol	Improved
Vidula et al., 2021 [25]	60/F	BNT162b2	2^nd^	4	Chest pain, nausea	Elevated	< 50	Abnormal	NA	metoprolol succinate, lisinopril	Improved

Sixteen (75%) patients were female, and four (25%) patients were male, with a median age of 61.5 years (mean ± SD: 55.81 ± 19.13), and the median number of days to onset of the first symptom was 3 (mean ± SD: 3.12 ± 2.24). Length of hospital stay was not reported in five patients, and the mean length of hospital stay among 67% of patients was 7.27 ± 3.95 (Table 3).

**Table 3:** Demographics of patients included in the study SD: standard deviation

Variable	Mean	SD
Age (years)	55.81	19.13
Days to first symptom onset	3.12	2.24
Length of hospital stay (days)	7.27	3.95

Among the COVID-19 vaccine types, 87.5% of the patients received mRNA COVID-19 vaccine, and 13% received a viral vector vaccine (Table 4). Regarding COVID-19 vaccine dose-induced TCM, 43.5% of patients reported TCM with the first dose regardless of the type of vaccine (Table 5). Chest pain was the most underlined manifestation (62.5%) in individuals with COVID-19-induced TCM, followed by dyspnea (31.25%), nausea (18.75%), and atypical symptoms (25%). Two patients reported no symptoms (Table 6).

**Table 4:** Types of COVID-19 vaccines and their percentage

Vaccine type	Number of patients	Percentage (%)
Pfizer-BioNTech	8	50
Moderna	6	37.5
ChAdOx1 nCoV-19	2	12.5

**Table 5:** Dosage of COVID-19 vaccine inducing TCM TCM: takotsubo cardiomyopathy

Dose of COVID-19 vaccine	Number of patients	Percentage (%)
1^st^	7	43.75
2^nd^	7	43.75
3^rd^	1	6.26
No dose reported	1	6.26

**Table 6:** Clinical manifestation of patients presented with COVID-19 vaccine-induced TCM TCM: takotsubo cardiomyopathy

Clinical symptomology	Number of patients	Percentage (%)
Chest pain	10	62.5
Dyspnea	5	31.25
Nausea	3	18.75
Atypical	4	25

All patients were found to have elevated cardiac troponin with abnormal ECG findings and abnormal LVEF on echocardiography (Table 7). Fourteen (87.5%) patients showed successful recovery and were discharged, and 12.5% of the patients died.

**Table 7:** Cardiovascular parameters of the patients presented with TCM ECG: electrocardiography; TCM: takotsubo cardiomyopathy

Variable		Number of patients	Percentage (%)
Cardiac troponin	Elevated	16	100
	normal	0	0
ECG findings	ST-segment changes	5	31.25
	T-wave changes	8	50
	Prolonged QTc interval	3	18-75
Ejection fraction (%)	< 50	15	92
	> 50	1	6.25
Outcome	Discharge	14	87.5
	Died	2	12.5

Discussion

The study's demographic profile reveals that most patients (75%) were female, with a median age of 61.5 years. This distribution aligns with the general epidemiology of TCM, which predominantly affects postmenopausal women. The mean time to symptom onset following COVID-19 vaccination was 3.12 days, emphasizing the acute nature of the cardiac complications [13]. Chest pain emerged as the predominant symptom, reported in 62.5% of patients. This aligns with the typical presentation of TCM, where acute emotional or physical stressors trigger severe chest pain, mimicking symptoms of myocardial infarction [15]. Additionally, dyspnea, nausea, and atypical symptoms were also observed, highlighting the diverse clinical spectrum of TCM [17].

The analysis of the included cases indicates that TCM occurred after both the first and second doses of COVID-19 vaccines, with 43.75% of patients experiencing TCM following each dose. Examining the dosing of COVID-19 vaccines in the context of TCM, it is noteworthy that 43.75% of patients experienced TCM after the first dose, irrespective of the vaccine type. This finding challenges the assumption that TCM might be associated solely with the second dose, as observed in some vaccine-related adverse events [26]. Jani et al. reported that COVID-19 vaccine-induced TCM had been reported after administration of the first or second dose [20]. Similarly, a 30-year-old female presented with sudden-onset dyspnea and chest pain following the second dose of the COVID-19 vaccine and was diagnosed with TCM [15]. This temporal association with vaccination provides the potential link between the immune response triggered by the vaccine and the development of TCM, which needs to be further studied.

Our study reveals that the majority (87.5%) of patients who developed TCM received mRNA COVID-19 vaccines, specifically Pfizer-BioNTech and Moderna. This observation raises intriguing questions about potential mechanisms specific to mRNA vaccines that might contribute to the development of TCM [27]. The exact mechanisms linking mRNA vaccines to TCM remain elusive and warrant further investigation. The study's outcomes shed light on the severity and recovery of patients who developed TCM post-vaccination. Among the cohort, 87.5% of patients were discharged following successful recovery, emphasizing the transient and reversible nature of TCM. However, 12.5% of patients developed the complication, underscoring the potentially life-threatening nature of this vaccine-related adverse event.

The specific mechanisms underlying vaccine-induced TCM remain unclear. It has been proposed that certain changes in ischemia-induced myocardium may induce TCM triggered by certain potential factors, including microvessel dysfunction, coronary artery vasospasm, and direct myocardial injury due to surge currents [28,29]. This phenomenon is triggered by the excessive release of catecholamines, including epinephrine or norepinephrine, through marked activation of the sympathetic nervous system (SNS), which may involve the hypothalamic-pituitary-adrenal axis [30]. Impaired neural networks in the limbic region may also be impaired during stress in patients with TCM [30]. A rat model of TCM suggests that epinephrine affinity switching from beta-2-adrenoceptors-Gs to Gi protects the myocardium from toxicity during stress [31]. Studies have reported elevated cytokine levels and inflammatory myocardial macrophage infiltration in TCM patients. Endothelial dysfunction in TCM patients is also a documented phenomenon [32]. Similarly, another potential mechanism involves the free-floating spike protein interaction with angiotensin-converting enzyme-2 (ACE-2) receptors, leading to an imbalance between angiotensin II and angiotensin, possibly leading to an acute elevation in blood pressure and myocardial stress following vaccination [33]. Acute emotional or physical stress may induce massive release of cortisol and catecholamines, stimulating various pathways, including coronary artery spasm, direct myocardial cell injury, and microvascular dysfunction [34].

Ongoing surveillance and reporting of vaccine-related adverse events, including TCM, are crucial for refining risk-benefit assessments and optimizing vaccination strategies. Establishing standardized diagnostic criteria and management guidelines for vaccine-induced TCM is imperative to ensure timely and effective interventions [14]. Future research should focus on elucidating the specific mechanisms linking COVID-19 vaccination, especially mRNA vaccines, to the development of TCM. Large-scale epidemiological studies, genetic analyses, and in-depth exploration of the immune response post-vaccination are essential to unravel the complexities of this rare adverse event [27].

Despite its valuable insights, the study has limitations that must be acknowledged. The small sample size of 16 patients from 15 case reports limits the generalizability of findings. Additionally, reporting biases and the potential for underreporting or selective reporting of cases may impact the overall interpretation of the results. The absence of a control group further limits the ability to establish a direct causal relationship between COVID-19 vaccination and TCM. Moreover, we do not have any data on the patients regarding previous history of COVID-19 infection or ischemic heart disease.

## Conclusions

This review contributes valuable insights into the rare but severe complication of TCM following COVID-19 vaccination and has significant clinical implications. The study emphasizes the need for continued surveillance, in-depth research, and standardized reporting of vaccine-related adverse events. Clinicians should remain vigilant, considering TCM in the differential diagnosis of patients presenting with chest pain or dyspnea post-vaccination, especially in the context of mRNA vaccines. As the global community strives to balance the benefits and risks of vaccination, ongoing research is essential to refine guidelines and optimize the safety profile of COVID-19 vaccines.
